# Prevalence Distribution and Risk Factors for *Schistosoma hematobium* Infection among School Children in Blantyre, Malawi

**DOI:** 10.1371/journal.pntd.0000361

**Published:** 2009-01-20

**Authors:** Atupele P. Kapito-Tembo, Victor Mwapasa, Steven R. Meshnick, Young Samanyika, Dan Banda, Cameron Bowie, Sarah Radke

**Affiliations:** 1 Department of Epidemiology, Gillings School of Global Public Health, University of North Carolina, Chapel Hill, North Carolina, United States of America; 2 Ministry of Health and Population, Blantyre District Health Office, Blantyre, Malawi; 3 Department of Community Health, University of Malawi College of Medicine, Chichiri, Blantyre, Malawi; 4 Department of Microbiology, University of Malawi College of Medicine, Chichiri, Blantyre, Malawi; George Washington University, United States of America

## Abstract

**Background:**

Schistosomiasis is a public health problem in Malawi but estimates of its prevalence vary widely. There is need for updated information on the extent of disease burden, communities at risk and factors associated with infection at the district and sub-district level to facilitate effective prioritization and monitoring while ensuring ownership and sustainability of prevention and control programs at the local level.

**Methods and Findings:**

We conducted a cross-sectional study between May and July 2006 among pupils in Blantyre district from a stratified random sample of 23 primary schools. Information on socio-demographic factors, schistosomiasis symptoms and other risk factors was obtained using questionnaires. Urine samples were examined for *Schistosoma hematobium* ova using filtration method. Bivariate and multiple logistic regressions with robust estimates were used to assess risk factors for *S. hematobium*. One thousand one hundred and fifty (1,150) pupils were enrolled with a mean age of 10.5 years and 51.5% of them were boys. One thousand one hundred and thirty-nine (1,139) pupils submitted urine and *S. hematobium* ova were detected in 10.4% (95%CI 5.43–15.41%). Male gender (OR 1.81; 95% CI 1.06–3.07), child's knowledge of an existing open water source (includes river, dam, springs, lake, etc.) in the area (OR 1.90; 95% CI 1.14–3.46), history of urinary schistosomiasis in the past month (OR 3.65; 95% CI 2.22–6.00), distance of less than 1 km from school to the nearest open water source (OR 5.39; 95% CI 1.67–17.42) and age 8–10 years (OR 4.55; 95% CI 1.53–13.50) compared to those 14 years or older were associated with infection. Using urine microscopy as a gold standard, the sensitivity and specificity of self-reported hematuria was 68.3% and 73.6%, respectively. However, the positive predictive value was low at 23.9% and was associated with age.

**Conclusion:**

The study provides an important update on the status of infection in this part of sub-Saharan Africa and exemplifies the success of deliberate national efforts to advance active participation in schistosomiasis prevention and control activities at the sub-national or sub-district levels. In this population, children who attend schools close to open water sources are at an increased risk of infection and self-reported hematuria may still be useful in older children in this region.

## Author Summary

*Schistosoma hematobium* infection is a parasitic infection endemic in Malawi. Schistosomiasis usually shows a focal distribution of infection and it is important to identify communities at high risk of infection and assess effectiveness of control programs. We conducted a survey in one district in Malawi to determine prevalence and factors associated with *S. hematobium* infection among primary school pupils. Using a questionnaire, information on history of passing bloody urine and known risk factors associated with infection was collected. Urine samples were collected and examined for *S. hematobium* eggs. One thousand one hundred and fifty (1,150) pupils were interviewed, and out of 1,139 pupils who submitted urine samples, 10.4% were infected. Our data showed that male gender, child's knowledge of an existing open water source (includes river, dam, springs, lake, etc.) in the area, history of urinary schistosomiasis in the past month, distance of less than 1 km from school to nearest open water source and age 8–10 years compared to those 14 years and older were independently associated with infection. These findings suggest that children attending schools in close proximity to open water sources are at increased risk of infection.

## Introduction

Schistosomiasis remains an important public health problem globally with an estimated 200 million cases reported each year [1]. However, 85% of the cases reported annually occur in sub-Saharan Africa and over 150,000 deaths are attributable to chronic infection with *S. haematobium* in this region [2],[3]. The eggs of *S. haematobium* provoke granulomatous inflammation, ulceration, and pseudo-polyposis of the vesical and ureteral walls. Hematuria is a very common sign of infection but other signs include dysuria, pollakisuria, and proteinuria. Kidney failure deaths due to urinary tract scarring, deformity of ureters and the bladder caused by *S. haematobium* infection have become less common due to modern drugs [4],[5]. Subtle and indirect morbidities such as fatigue, physical or cognitive impairment and effects of co-infections with other infectious diseases like HIV, malaria have received more attention recently [4]. New evidence from a recent review of these studies suggests a causative link between schistosome infection, anti-parasite inflammation, and risk for anaemia, growth stunting and under-nutrition, as well as exacerbation of co-infections and impairment of cognitive development and physiological capacities among infected individuals [6]. The causal relationship between anaemia and schistosomiasis exists even after controlling for other co-infections and dietary factors among pregnant women and children [6]–[9]. The underlying mechanisms proposed range from social determinants to complex immune interactions.

In Malawi, schistosomiasis is endemic with *S. haematobium* being highly prevalent in the southern region while *S. mansoni* predominates on the central plain and the northern regions [10]. The national schistosomiasis control program estimates that between 40% and 50% of the total Malawian population is infected with schistosomiasis (National Plan of Action for the Control of Schistosomiasis and Soil transmitted Helminthes five-year plan 2004–2008). Other studies have suggested that these national estimates may have been derived from studies conducted years ago that had some selection bias for high risk schools [11]. A national survey conducted in 2002 among primary school pupils found the prevalence of *S. hematobium* and *S. mansoni* infection among school children to be 6.9% and 0.4% using filtration method of urine and Kato-Katz method for stools respectively [11]. These findings were much lower than expected and it was concluded that schistosomiasis is highly localized in Malawi. This implies that local estimates would be more useful than national estimates in guiding the selection of control strategies to be implemented at district or sub-district level which depends on disease prevalence rate in a community [12]. However, most districts in Malawi do not have local estimates to guide planning and implementation of control interventions at that level. The Ministry of Health through the national schistosomiasis control program is currently undertaking a deliberate effort to have schistosomiasis prevention and control efforts integrated within district plans to encourage ownership and improve sustainability by engaging district teams (National Plan of Action for the Control of Schistosomiasis and Soil transmitted Helminthes five-year plan 2004–2008).

Blantyre district health team initiated and conducted this study aimed at determining the prevalence of schistosomiasis as part of this effort. The findings are expected to be used as baseline for future evaluation and monitoring of control activities. A representative cross-sectional study of urinary schistosomiasis infection among school children in Blantyre district was therefore conducted. To establish the need for intervention in a community, we conducted our baseline survey among children sampled from grade three in elementary schools as it has been shown that this population is intensely affected in endemic communities [13]. The prevalence distribution, factors associated with *S. hematobium* infection and the reliability of self reported hematuria compared to the “gold standard” parasitological examination among school children are described.

## Materials and Methods

### Study area

This cross-sectional study was carried out in Blantyre district located in southern Malawi. The district has a land area of 2,012 km^2^ and the altitude above sea level ranges from 300 m to 1000 m. According to the national statistical office, it has an estimated population of one million of whom 61% reside in the urban or peri-urban areas.

**Figure 1:**
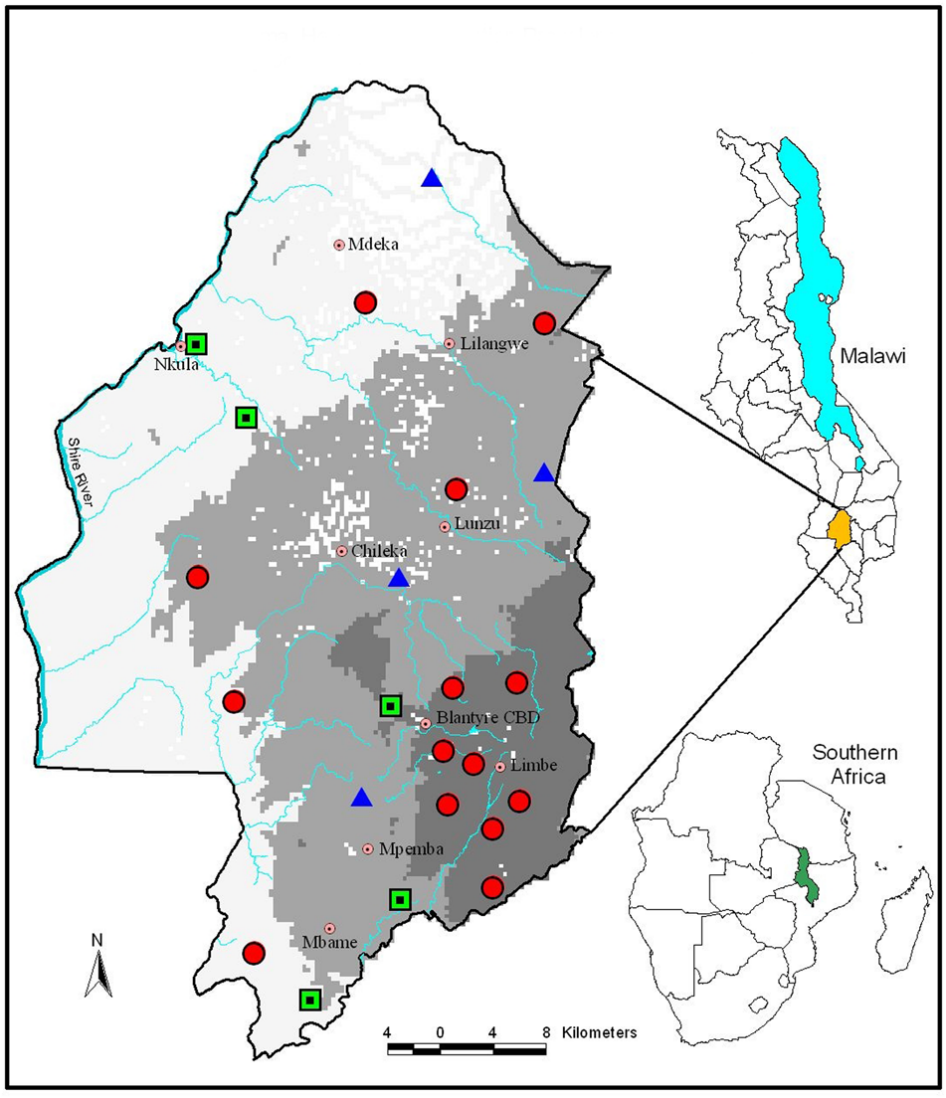
Prevalence distribution of *Schistosoma hematobium* infection in Blantyre district, Blantyre Schistosomiasis survey 2006, Malawi. Prevalence (%): 0–9 (large red circle), 10–20 (blue triangle), >21 (green square). Altitude (metres): lowest–500 (white), 500–1000 (light grey), >1000 (dark grey). Main centres are indicated by small red dots and main rivers by blue lines.

From north-west to south-west boundary of the district runs the Shire River which is the main outlet of Lake Malawi (Figure 1). The average annual temperature is 27°C whilst the average rainfall is 871 mm. The rainy season is from November to April and the dry season from May to October [14].

### Sample selection

All standard three pupils who were attending primary schools in Blantyre district during the time of data collection between May and July 2006 were eligible to participate in our study. Our study sample was selected using a stratified 2-stage probability sampling technique. First, we stratified the district was into 3 ecological risk areas for urinary schistosomiasis which was based on altitude above sea level as follows; high (<500 m), moderate (500–1000 m) and low (>1000 m). Some previous studies have shown that altitude above sea level is known to be associated with schistosomiasis [15],[16]. We used STATA software v10.1 (StataCorp Ltd, Texas, USA) to estimate sample size for comparison of proportions between strata. The sample size of 1,128 was estimated using a prevalence estimate of 40.0% with 80.0% power to detect a relative difference of 10.0% between strata at a 0.05 level of significance. Second we obtained from the district education offices the number of schools and pupils' population estimates in each stratum. Based on the pupils' population distribution within the strata and our prior decision to randomly select 50 standard three pupils from each selected school, we calculated that 23 schools would be required. Studies have shown that Lot-Quality Assurance Scheme approaches provide the ability to identify communities with a high prevalence of schistosomiasis with high levels of sensitivity and specificity, even at very small maximum sample sizes [17]. Finer classification of schools according to categories of prevalence are achieved with moderate sample sizes of ≥15 and have been found to be more accurate with extremely low probabilities of making gross classification errors. Thirdly, sampling was conducted in 2 stages. In the first stage, the primary sampling units were schools that were selected with a probability proportional to number of schools in the strata. A list of schools in each stratum was compiled and using computer generated random numbers schools in each stratum were selected as follows; six schools from the high risk stratum, eight from the moderate risk stratum and nine from the low risk stratum. In the second stage, a random sample of 50 grade three pupils in each selected school was obtained.

### Data collection

Children were interviewed by trained Health Surveillance Assistants (HSAs) and community health nurses using a questionnaire that was adapted from the 2002 national prevalence survey [11]. To reduce bias and improve the performance of the questionnaires, questions about schistosomiasis were disguised among other health related questions. The questionnaire was pre-tested and modifications were made after discussions with HSAs, teachers and district health office staff. Risk factors included main household water source, child's knowledge of nearby open water sources (open water source defined as any open water body including lakes, springs, rivers, streams, ponds, swamps and dams), frequency of contact with open water sources, dysuria or passing blood in urine within the past month, history of *S. hematobium* infection and *S. hematobium* treatment. Other factors included urban or rural location, proximity of school to an open water source and household socio-economic status (SES).

We used a method developed by the Centre for Social Research in Malawi (Kadzandira et al. unpublished report) and similar methods have been used in other studies to estimate SES [18]. The method combined six variables to formulate the complex indicator of SES which includes housing structures, main occupation of heads of households, and possession of selected assets such as radios, telephones, televisions, etc. For each variable, households were assigned a weight ranging from zero to two. For example, households with a grass-thatched roof were given a weight of zero while households with a plastic paper roof were given a 0.3 and households with iron-sheet or tiled roof were given a 1.0 (Table S1). The sum of weights from the six variables determined the SES score of each household. Households were then classified as low SES for those with scores less than 4.0, moderate SES for 4.0–6.0 and high SES for more than 6.0. There were no significant differences when we analyzed our data using the year 2000 World Bank asset scoring system for wealth (data not shown) [19].

In addition to the questionnaire interview, pupils were asked to submit urine in supplied 20 ml screw top plastic containers between 0900 hrs and 1400 hrs. The samples were immediately transported to the University of Malawi College of Medicine microbiology laboratory. Our primary outcome was *S. hematobium* infection as diagnosed by urinary microscopy examination.

### Laboratory analysis

Samples were tested for micro-hematuria using urine reagent strips (Uripath, Plasmatec Laboratory, UK) and results were scored as negative, +, ++ or +++ as per manufacturer recommendations. 10 mls of urine was filtered using paper filters (Gelman Sciences, Michigan USA) and the egg count was recorded per 10 mls of urine. In close collaboration with the district health office, all children who were found to be positive either through microscopic examination or urine reagent strips were referred for treatment.

### Data analysis

Data were entered into Microsoft Access 2003. The data were then converted into SAS version 9.1 (SAS Institute, Cary, North Carolina, USA) which was used for all analyses. Based on the study design of the survey; weighting, stratification, and clustering were taken into account in all statistical analyses using Survey procedures in SAS. The survey analysis procedures use the Taylor series expansion method to estimate sampling errors of estimators based on sample designs [20]. The procedures estimate the variance from the variation among the schools and pool stratum variance estimates to compute the overall variance estimate. A weighting factor was used in the analysis to reflect the likelihood of sampling each student in a stratum. The weight used for estimation is given by the following formula:
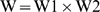
Where W1 = the inverse of the probability of selecting the school in a stratum and, W2 = the inverse of the probability of selecting a child from the classroom within the school. Bivariate analyses were used to estimate the crude odds ratios and identify variables to be included in the initial multivariate logistic regression model. Chi-square test was used to assess the association between categorical variables and *S. hematobium* infection in bivariate analysis. Variables that had a p value <0.20 were included in the initial multivariate logistic regression model. Using backward elimination method, variables that showed independent association with *S. hematobium* infection at a significance level of p value <0.05 were retained in the model. A significance level of p value <0.05 was used in all other analyses. Pearson correlation coefficient was used to test for correlation between continuous variables.

### Ethical considerations

The study was conducted using protocols approved by the institutional ethics Review Boards of the University of North Carolina at Chapel Hill, USA and the University of Malawi, College of Medicine.

Prior to conducting the study, aims and procedures to be used to collect data were explained to parents or guardians and community leaders including school committees during meetings. Written consent was obtained from the local leaders, children's parents or guardians and assent was subsequently obtained from the children.

## Results

From a total of 195 schools, 23 schools were selected. A total of 1,150 pupils were interviewed and 1,139 pupils submitted urine specimen which was examined for *S. hematobium* ova. 4 pupils (0.35%) refused to submit urine specimen and 7 pupils (0.60%) reported that either they did not have the urge to produce urine during the time the study team was at the school or they submitted urine specimen of less than 10 mls.

### Characteristics of the study population

The mean age of the study population was 10.5 years and most of the children (86.2%) were aged 8 to 13 years with no significant difference by gender. About 44.2% were from poor SES while 21.9% of the children were from high socio-economic households. Characteristics of the study sample are shown in Table 1.

**Table 1:** General characteristics of school children, Blantyre Urinary Schistosomiasis Survey, Malawi, 2006.

Characteristic	N	Percentage§
Gender*		
Male	590	51.53
Female	555	48.47
Age*		
≤7 yrs	63	5.52
8–10 yrs	563	49.34
11–13 yrs	420	36.81
≥13 yrs	95	8.33
Location		
Rural	700	60.87
Urban	450	39.13
Stratum		
Shire river/altitude <500 m	300	26.09
Altitude 500 m–1000 m	400	34.78
Altitude >1000 m	450	39.13
Socio-economic status		
Low	508	44.17
Moderate	390	33.91
High	252	21.91

**§:** Not accounted for clustering, stratification and not weighted.

*: Gender and age were missing for 5 and 9 children respectively.

### Prevalence and distribution of infection

*S. hematobium* eggs were found in 10.4% (95% confidence interval (CI): 5.4–15.4%) of the pupils who submitted urine specimen. Prevalence in schools ranged from 0.0% to 46.0% and 39.1% (9/23) of the schools had infection prevalence of ten percent or more (Figure 1). Prevalence varied by altitude above sea level; 17.8% of children from <500 m stratum had positive results for *S. hematobium* ova, 12.4% in the 500–1000 m stratum and 3.60% in the >1000 m stratum (p = 0.007).

**Figure 2:**
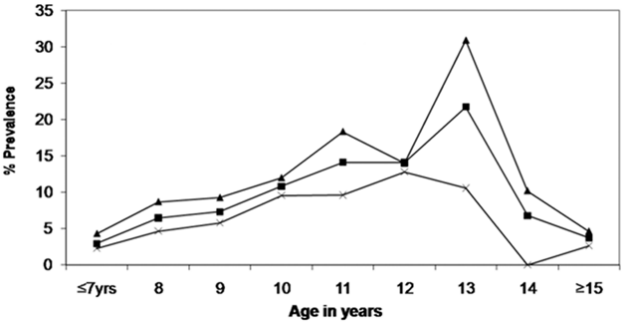
Prevalence of *Schistosoma hematobium* infection by age and sex, Blantyre Schistosomiasis survey 2006, Malawi. Overall (square), male (triangle), female (×).

Infection was higher in rural areas at 14.4% (95% CI: 6.6–22.2%) compared to the urban areas 3.6% (95% CI: 1.4–5.8%). 13.2% of the boys in the study population were infected compared to 7.4% among the girls. Across all age ranges, boys had higher prevalence compared to girls (Figure 2). The prevalence showed an increasing trend with increasing age and peaked around 11–13 years then started to decline.

Boys were more likely to be knowledgeable of existence of open water source in their area compared to girls, 61.8% and 55.0% respectively (p = 0.03). Among pupils who were knowledgeable of an open water source in their area, children aged 14 years or older were less likely to report playing or swimming in water compared to those less than 14 years old, 79.1% and 90.8% respectively (p = 0.003). There was no significant difference in the proportions that reported daily/ weekly water contact frequency among boys and girls who reported playing or bathing in open water sources, 85.6% and 88.1% respectively (p = 0.4). Children who reported previous history of urinary schistosomiasis infection were more likely to report ever being treated for urinary schistosomiasis in the past compared to children who reported no previous history of schistosomiasis (27.1% and 3.7%; p<0.001).

### Intensity of infection

The overall ova density (eggs/10 ml of urine) among the infected in the study population was 10.1 (range 1–80). The prevalence of heavy infection (≥50 eggs/10 mls) among those infected was 2.4%. The average ova density was calculated for each school and was compared with school prevalence. There was a strong correlation between prevalence of *S. hematobium* infection and mean ova density in schools (Pearson correlation coefficient r = 0.65 p = 0.005). There was no correlation between age and ova density among those infected. There was no difference in the mean ova density between boys and girls (mean; 9.9 and 10.4 eggs/10 mls urine p = 0.8).

### Accuracy of self reported hematuria

Of the 1,150 pupils interviewed, 353 (31.6%, 95%CI: 23.1–40.1%) reported passing blood in urine (hematuria) over the past month. Proportion of pupils reporting passing blood in urine ranged from 8% to 70% in schools. Using urine microscopic examination as a gold standard, the sensitivity and specificity of self-reported hematuria was 68.3% and 73.6% respectively. The negative predictive value (NPV) was 95.0% while positive predictive (PPV) value was very low at 23.9%. PPV of self-reported hematuria was higher among boys compared to girls (27.3% versus 18.4%) and was higher among pupils aged 9 years or more compared to those aged 8 years old or less (17.0% versus 24.8%).

### Factors associated with urinary schistosomiasis

Table 2 shows the association between risk factors and *S. hematobium* infection in bivariate analyses. The following factors were significantly associated with infection in bivariate analysis; age 11–13 years, socio-economic status, gender, location, household water source, pupil's knowledge of existence of an open water source, previous history of urinary schistosomiasis and proximity of school to an open water source. Distance from home to an open water source, previous history of schistosomiasis treatment, frequency of water contact and dysuria were not significantly associated with infection. In multivariate analysis, factors that remained significantly associated with *S. hematobium* infection were male gender (OR 1.81; 95% CI 1.06–3.07), child's knowledge of an open water source in the area (OR 1.90; 95% CI 1.14–3.46), previous history of urinary schistosomiasis (OR 3.65; 95% CI 2.22–6.00) distance of less than 1 km from school to nearest open water source (OR 5.32; 95% CI 1.66–17.07) and age 8–13 compared to those age 14 and older (Table 3).

**Table 2:** Bivariate analysis of factors associated with *Schistosoma hematobium*, Blantyre schistosomiasis survey, Malawi, 2006.

Risk factor	N	No. infected†	OR (95% CI)‡
Age			
≥14 yrs	92	6	1
≤7 yrs	63	2	0.57 (0.52–6.19)
8–10 yrs	562	46	1.75 (0.65–4.72)
11–13 yrs	414	69	3.43 (1.35–8.68)
Socio-economic status			
High	249	6	1
Moderate	388	47	3.56 (1.11–11.42)
Low	502	70	4.56 (1.25–16.61)
Gender			
Female	547	42	1
Male	587	80	1.89 (1.10–3.23)
Main household water source			
Piped water	437	28	1
Borehole	493	64	2.13 (0.79–7.74)
Wells/River/Streams/Dams/springs	206	30	2.63 (1.07–6.46)
Location			
Urban	444	19	1
Rural	695	104	4.50 (1.95–10.39)
Knowledge of open water source			
No	474	17	1
Yes	657	106	4.43 (2.91–6.76)
Distance from home to open water source*			
Far/ Very far	138	22	1
Close/ Very close	509	78	1.09 (0.63–1.89)
Play/bath in open water source*			
No	69	10	1
Yes	580	95	1.21 (0.35–4.10)
Frequency of open water source contact*			
>Monthly	74	12	1
Daily/Weekly	505	83	1.18 (0.58–2.40)
History of dysuria			
No	874	77	1
Yes	250	46	1.97 (0.89–4.34)
History of schistosomiasis			
No	830	50	1
Yes	303	73	4.16 (2.35–7.35)
History of schistosomiasis treatment			
No	1023	104	1
Yes	112	19	1.61 (0.74–3.49)
Distance from school to nearest open water source			
≥1 km	696	25	1
<1 km	443	98	5.52 (2.12–14.39)

*: Base population are the 657 pupils with knowledge of open water source.

**†:** Infection characterized by parasitological examination of urine.

**‡:** Odds ratios and 95% confidence intervals are weighted and account for stratification and clustering.

**Table 3:** Adjusted association of *Schistosoma hematobium* by risk factor, Blantyre schistosomiasis survey, Malawi, 2006*.

Risk factor	OR (95% CI)†	p-value
Age		
≥14 yrs	1	
≤7 yrs	2.34 (0.24–22.43)	0.6
8–10 yrs	4.55 (1.53–13.50)	0.007
11–13 yrs	4.27 (1.49–12.23)	0.007
Gender		
Female	1	
Male	1.81 (1.06–3.07)	0.03
Knowledge of an open water source		
No	1	
Yes	1.90 (1.14–3.46)	0.04
History of schistosomiasis		
No	1	
Yes	3.65 (2.22–6.00)	<0.0001
Distance from school to nearest open water source		
≥1 km	1	
<1 km	5.39 (1.67–17.42)	0.005
Socio-economic status		
High	1	
Medium	2.33 (0.68–8.00)	0.2
Low	1.72 (0.44–6.72)	0.4

*: Adjusted for age, gender, open water source knowledge, history of schistosomiasis, distance from school to nearest open water source, location, socio-economic status, dysuria, history of schistosomiasis treatment and household water source.

**†:** Odds ratios and 95% confidence intervals are weighted and account for stratification and clustering.

## Discussion

To our knowledge this is the first large-scale survey that has been conducted in Blantyre district to determine prevalence and risk factors associated with *S. hematobium* infection. The prevalence of *S. hematobium* infection was 10.4% and ranged from 0.0% to 46% in schools in our study. Male gender, age, child's knowledge of an open water source in the area, previous history of schistosomiasis and proximity of school to an open water source were independently associated with infection in our study.

The last published *S. hematobium* prevalence estimates for the district were from studies conducted between 1979 and 1981 among pupils from three schools and individuals from two villages where prevalence ranged from 22% to 72% using centrifugation and reagent dipsticks respectively [10]. Our study prevalence estimate is lower than the national estimates of between 40% and 50% currently in use. Our prevalence findings are similar to the national schistosomiasis survey results [11]. The observed differences between the national estimates in current use and the recent observed findings have been attributed to effective schistosomiasis control and availability of anti-schistosomiasis drugs [11]. In addition the differences in size and methodology of studies could partly explain the observed findings. The more recent large-scale surveys are likely to be more accurate compared to previous studies that may have had some selection bias for high risk schools [11]. Also, the wide range of infection prevalence rates among schools in our study illustrates the focal distribution characteristic of schistosomiasis.

The association between male gender and *S. hematobium* found in our study is similar to findings from other studies [21]–[23]. Boys were more likely to be infected and be knowledgeable of the existence of an open water source in their area compared to girls. In other studies, boys had more water-contact compared to girls [23]. This could be partly explained by the fact that boys are usually more adventurous, they are more likely to be knowledgeable of their environments including water bodies and therefore be more likely to play in them compared to girls. The association between gender and *S. hematobium* infection varies in different communities. Some studies have reported no association between *S. hematobium* infection and gender [22],[24] while other studies have reported association with female gender [25].

Our study showed that there was an increasing trend of infection among children from six years to thirteen years with a decline from 14 years. Also, children aged 14 years or more were less likely to report playing or swimming in water in our study. This suggests that children 14 years and older have lower risk of being infected as they are less likely to be engaged in recreational water-contact behaviors compared to younger children. Studies conducted elsewhere have reported similar results, for example, throughout the nine year schistosomiasis study in Kenya, schistosomiasis infection was lower in older children [22],[26]. Other studies have reported that age-acquired immunity to re-infection contributes to the declining trend in prevalence among children aged 15 years and older [27]. However, we cannot conclude whether age-acquired immunity contributed to our findings since we did not find a negative association between ova load and age.

School proximity to an open water source showed a very strong association with infection. Proximity to water sources has been consistently shown to be associated with schistosomiasis infection in other studies [23],[28],[29]. In contrast, other studies have found no association or variable influence on schistosomiasis infection and it has been suggested that multiple uses of various water bodies with different transmission levels could explain these findings [30],[31]. Interestingly, we did not find a significant association between proximity of household to an open water source in our study. This could be partly explained by the following reasons; first, 95.0% of pupils who were infected and reported that their homes were far or very far from an open water source attended schools that were less than 1 km from an open water source. This means that while children may not engage in water contact recreational activities close to their homes, they might have been exposed when going or coming from school or other places away from home. Secondly, our questionnaire method of estimating distance from home to nearest water source was more subjective and prone to misclassification. Hence our estimate for the effect of household proximity to water source on infection could have been biased.

Communities with high infection rates are usually clustered around contaminated water sources [32],[33]. This could partly explain why we observed an association between *S. hematobium* infection and previous history of urinary schistosomiasis since children who reported previous history of schistosomiasis were more likely to report ever being treated for urinary schistosomiasis. We did not find a significant association with reported water contact frequency. Studies have found variable influence of water contact frequency on schistosomiasis infection. This has been attributed to variability in amount of body exposed to water and others have suggested that other factors play major role [22],[34].

Socioeconomic status of the household was not an independent factor associated with infection in our study population. Conflicting results have been reported in previous studies [35]–[37]. Based on our findings, possibly improving socioeconomic status alone may not significantly reduce the rate of infection among this population.

The performance of self reported hematuria in our study is similar findings from other endemic countries with sensitivity and specificity range of 50–100% and 58–96% respectively in moderate and high transmission areas [38]–[40]. Our positive predicted value of self-reported hematuria was low and could be due to several reasons. First, our study was limited since only one urine specimen was collected and we did not encourage the children to conduct exercises prior to urine collection. Studies have shown that repeated examination of urine specimen over consecutive days and exercises prior to urine collection improve egg detection [41],[42]. It is possible to have an infected child reporting hematuria with no ova detected one urine sample. Also, this could have been exacerbated by the fact that our study population had low infection intensity (10.1 eggs/10 mls urine). Our results probably could have been different if reported hematuria was compared to Circulating Anodic Antigen (CAA) from *S. haematobium*. Secondly, children that either reported passing blood in urine or tested positive for *S. hematobium* infection were referred for treatment and it might be possible that some children reported hematuria to be referred to health facilities and hence affecting accuracy.

This study was initiated and implemented by the district health office. It demonstrates the success of the persistent and deliberate national efforts advocating for active participation at the sub-national or sub-district level in schistosomiasis prevention and control activities in countries where schistosomiasis is endemic. This is crucial for long-term ownership and sustainability of schistosomiasis control efforts. *S. hematobium* infection was found to be localized in Blantyre district. The district health office in collaboration with the district education offices may consider targeted treatment every two years in schools and surrounding communities with prevalence of between 10% but less than 50% as recommended by WHO in addition to passive treatment in the health facilities and snail control [12]. Even though the predictive value for the self-reported hematuria was low, the sensitivity and specificity was comparable to other previous studies and suggests that the use of questionnaires in this part of Sub-Saharan Africa may still valuable in older children [43]. Further intervention studies to determine the best and cost-effective strategies to provide treatment to children and communities in the affected areas are required. Ecological studies are also needed to identify transmission foci to facilitate implementation of ecologically targeted control measures.

## Supporting Information

Table S1 Weights used to estimate household socio-economic status
(0.03 MB DOC)
