# Supplementary material for: Prevalence Distribution and Risk Factors for Schistosoma hematobium Infection among School Children in Blantyre, Malawi
Source: PLoS Negl Trop Dis. 2009 Jan 20;3(1):e361. doi: 10.1371/journal.pntd.0000361 (PMC2614474; doi:10.1371/journal.pntd.0000361)
Supplement: Table S1 — Weights used to estimate household socio-economic status (0.03 MB DOC) [file pntd.0000361.s001.doc]

| Table S1  Weights used to estimate household socio-economic status | | | |
| --- | --- | --- | --- |
| **Variable** | **Weight** | **Variable** | **Weight** |
| Roof materials  Grass  Plastic sheets  Iron sheets  Tiles | 0.0  0.3  1.0  1.0 | Toilet facility  None  Flush toilet  Pit latrine  San plat latrine | 0.0  2.0  1.0  1.6 |
| Water source  Piped water  Open well  Protected well  Borehole  Stream/river  Lake/pond | 1.0  0.2  0.7  0.7  0.0  0.0 | Floor material  Earth/mud  Dung  Wood planks  Tiles  Cement  Carpet | 0.4  0.2  1.0  1.0  1.0  1.0 |
| Occupation of head of household  None  Farming  Self employment/business  Salaried  Ganyu (Piece work) | 0.0  0.5  0.6  1.0  0.3 | Household assets  Electricity  Radio  TV/Telephone  Refrigerator  Bicycle  Motorcycle  Car/Truck | 0.4  0.4  0.6  0.6  0.4  0.6  1.0 |
